# Bioactivity and Regenerative Potential of Calcium Silicate-Based Endodontic Sealers on Human Periodontal Ligament Stem Cells: A Systematic Review of In Vitro Cellular Mechanisms

**DOI:** 10.7759/cureus.116629

**Published:** 2026-09-21

**Authors:** Fahad I Yaseen, Abdulaziz M Alribi, Yasser K AlGhadyan, Nawaf A Alshehri, Thamer M Alanazi, Abdullah S Aldhafeeri, Marwan S Alyousef, Shaykha S Elkherayef, Abdulrahman A Albarqi, Abdulrahman M Alqahtani, Ali M Falatah

**Affiliations:** 1 Dentistry, Majmaah University, Riyadh, SAU; 2 Dentistry, King Saud bin Abdulaziz University for Health Sciences, Riyadh, SAU; 3 Dentistry, Vision College, Riyadh, SAU; 4 General Dentistry, Security Forces Hospital Dammam, General Administration of Medical Services, Ministry of Interior, Dammam, SAU; 5 Endodontics, King Fahd Hospital, Madinah Health Cluster, Madinah, SAU

**Keywords:** calcium silicate-based sealers, endodontic sealers, periapical healing, periodontal ligament stem cells, regenerative endodontics

## Abstract

Calcium silicate-based endodontic sealers are bioactive obturation materials that may support periapical regeneration, yet in vitro evidence remains fragmented and methodologically heterogeneous. This review evaluated whether these sealers show consistent cellular bioactivity in human periodontal ligament stem cells (hPDLSCs) and which outcomes and formulations show the most favourable regenerative potential. The review followed a predefined methodology informed by the Cochrane Handbook and was reported according to the Preferred Reporting Items for Systematic Reviews and Meta-Analyses (PRISMA) 2020. PubMed/MEDLINE, Scopus, Web of Science, the Cochrane Central Register of Controlled Trials, and Google Scholar were searched using Population, Intervention, Comparison, Outcomes, and Study design-based strategies. Risk of bias was assessed with the National Toxicology Program - Office of Health Assessment and Translation (NTP-OHAT) tool. Because materials, extract dilutions, exposure periods, assay protocols, and effect measures varied substantially, a structured narrative synthesis was undertaken instead of meta-analysis.

Of 891 records, 15 studies met the eligibility criteria. Most used primary hPDLSCs characterised by mesenchymal stem-cell markers and compared calcium silicate-based sealers, including TotalFill BC Sealer (FKG Dentaire Sàrl, Le Crêt-du-Locle, Switzerland)/EndoSequence BC Sealer (Brasseler U.S.A. Dental, LLC, Savannah, Georgia, USA), BioRoot RCS (Septodont, Saint-Maur-des-Fossés, France), CeraSeal (Meta Biomed Co., Ltd., Cheongju, Republic of Korea), Bio-C Sealer (Angelus, Londrina, Brazil), Well-Root ST (VERICOM Co., Ltd., Chuncheon, Republic of Korea), and NeoSEALER Flo (Avalon Biomed, Houston, Texas, USA), with epoxy resin-based sealers, predominantly AH Plus (Dentsply DeTrey GmbH, Konstanz, Germany). Overall risk of bias was low, although blinding of outcome assessment was often unclear.

Across 3-(4,5-dimethylthiazol-2-yl)-2,5-diphenyltetrazolium bromide (MTT) and CCK-8 assays, calcium silicate-based sealers generally maintained or increased cellular metabolic activity, whereas AH Plus showed lower activity; NeoSEALER Flo and EndoSeal MTA (Maruchi, Wonju, Republic of Korea) showed less favourable or intermediate responses. Twelve scratch-wound studies reported migration comparable with or greater than controls; eleven Alizarin Red S studies reported increased mineral deposition, most pronounced for TotalFill BC Sealer/EndoSequence BC Sealer and BioRoot RCS; and eleven reverse transcription quantitative polymerase chain reaction (RT-qPCR) studies reported upregulation of osteogenic and cementogenic markers, including ALP, RUNX2, CEMP1, and CAP. Six studies assessed inflammatory or immunomodulatory responses; most reported lower pro-inflammatory cytokine expression, particularly IL-6 and IL-8, with calcium silicate-based sealers than with AH Plus, although responses varied by formulation and exposure time. Within the limits of two-dimensional models and heterogeneous protocols, contemporary calcium silicate-based root canal sealers appear more cytocompatible and biologically active toward hPDLSCs than conventional epoxy resin sealers, supporting favourable cellular metabolic activity, migration, mineralisation, and osteogenic and cementogenic differentiation.

This review assessed whether calcium silicate-based sealers are cytocompatible and promote regenerative bioactivity in hPDLSCs.

## Introduction and background

Untreated caries in permanent teeth has been reported as the most prevalent condition worldwide, affecting approximately 2.4 billion people [1]. These findings highlight the substantial global burden of untreated dental caries [1]. Neglected dental caries may lead to pulpitis, which can subsequently result in periapical periodontitis, a common sequence of disease development [2].

Apical periodontitis (AP) is driven by persistent microorganisms harboured within the root canal system due to its intricate anatomy (including accessory canals, isthmuses, and dentinal tubules), despite thorough chemomechanical debridement [3]. The primary goal of endodontic treatment is to eliminate these microorganisms and seal the root canal system. Endodontic obturation (combining core materials and sealers) aims to trap residual microbes, prevent nutrient ingress due to apical/coronal leakage, and promote periapical repair [4].

Endodontic sealers are expected to exhibit radiopacity, minimal shrinkage, adherence to dentinal walls, low solubility and viscosity, suitable setting time, non-cytotoxicity, and other physicochemical properties [5]. Historically, sealers have been classified based on their chemical compositions as zinc oxide-eugenol, calcium hydroxide, glass ionomer, and epoxy resin sealers, and they were considered successful if they remained biologically indifferent to the surrounding tissues [6]. Torabinejad M et al. (1993) introduced mineral trioxide aggregate (MTA) to endodontics [7]. Bioceramic and calcium silicate-based sealers have shifted the endodontic paradigm from using bioinert materials to those emphasising bioactivity. Bioceramics are biocompatible ceramic materials or metal oxides, including alumina and zirconia, radiotherapy glasses, calcium silicates, hydroxyapatite, resorbable calcium phosphates, bioactive glass, and glass ceramics [8]. According to research findings, the biocompatibility and bioactivity of calcium silicate-based bioceramics are crucial in endodontics. Bioceramics affect the biological behaviour of immune cells, fibroblasts, osteoblasts, osteoclasts, and stem cells. However, the impact of bioceramic implantation on wound healing and tissue repair depends on the cellular response to the material [9]. They are designed to interact directly with biological systems, primarily through the release of calcium, hydroxide, and silicate ions, which facilitates the spontaneous formation of an apatite layer upon contact with phosphate-containing physiological fluids [10]. This apatite-containing interfacial layer has been proposed to improve adaptation and induce remineralisation by decreasing microleakage and increasing the hermetic seal [11]. Some common commercially available brands of calcium silicate-based sealers include iRoot SP (Innovative BioCeramix, Burnaby, British Columbia, Canada), EndoSequence BC Sealer (Brasseler U.S.A. Dental, LLC, Savannah, Georgia, USA), BioRoot RCS (Septodont, Saint-Maur-des-Fossés, France), MTA Fillapex (Angelus, Londrina, Paraná, Brazil), and EndoSeal MTA (Maruchi, Wonju, Republic of Korea) [12].

With advancements in biomaterials, standardised scientific terminology is important for meaningful interpretation and comparison across studies. Without an accurate operational definition of ‘bioactivity’, laboratory findings may be difficult to compare and interpret in relation to regenerative mechanisms. Estivalet MS et al. (2022) highlighted a divide in research approaches: acellular investigations often measure simple chemical reactivity (ion release and precipitate formation), whereas cellular studies focus on biological responses (enzymatic activity and gene expression), leading to conclusions and parameters that are difficult to compare across studies [13].

Thus, without a standardised framework, the biological relevance of ‘bioactivity’ remains difficult to interpret consistently across studies. Sanz JL et al. (2021) identified periodontal ligament stem cells (PDLSCs) as a biologically relevant cellular model for investigating mechanisms involved in periradicular repair [14]. The researchers monitored the enzymatic activity of alkaline phosphatase (ALP) as an early marker of mineralisation and used Alizarin Red staining (ARS) to quantify the formation of calcium nodules. The systematic review by Sanz JL et al. (2021) provides a critical cellular perspective, confirming that calcium silicate biomaterials generally demonstrate superior cytocompatibility compared to traditional resins [14]. However, a significant gap remains: excellent cytocompatibility (absence of overt cytotoxicity) is frequently conflated with true bioactivity (induction of differentiation). While the systematic review by Sanz JL et al. highlighted the potential of these materials to promote mineralisation, this potential has yet to be integrated with a standardised methodological mapping that accounts for the diverse testing variables observed in the field.

To investigate cellular mechanisms relevant to periapical repair, human periodontal ligament stem cell (hPDLSC)-based assays are particularly informative because these cells contribute to periodontal tissues, including cementum and alveolar bone. While the current literature is often fragmented across various cellular models, such as Saos-2 or human dental pulp cells (hDPCs) [15], a unified focus on PDLSCs allows for the evaluation of mechanistic proxies for repair [14].

The combination of methodological heterogeneity and material-specific differences supports the need for an integrated approach to interpreting this evidence. Greater standardisation of testing protocols may improve the comparability and translational relevance of in vitro findings.

A systematic review is therefore needed to synthesise the heterogeneous evidence, clarify the relationship between chemical testing and cellular mechanisms, and establish what current in vitro evidence supports regarding biological responses relevant to periapical repair. This systematic review aims to address this gap by synthesising the findings from in vitro studies on the bioactivity of calcium silicate-based sealers using hPDLSC models, including viability, migration, mineralisation, and osteogenic/cementogenic markers such as ALP, RUNX2, and CEMP1, through a structured narrative synthesis.

## Review

Designing a protocol and a search strategy for a database.

The review was conducted using a predefined systematic review methodology informed by the Cochrane Handbook for Systematic Reviews of Interventions [16] and was reported in accordance with PRISMA 2020 [17]. The main research question examined the cellular bioactivity of calcium silicate-based endodontic sealers in hPDLSCs. The PICOS framework was used to establish the eligibility criteria [18].

hPDLSCs or human periodontal ligament cells (hPDLCs) with documented stem/progenitor characteristics cultured in vitro were eligible. Primary cells were isolated from healthy human periodontal ligaments obtained from extracted teeth, usually third molars or premolars removed for orthodontic or surgical reasons, from donors aged 16-65 years. Stem/progenitor characteristics had to be confirmed by flow cytometry, including positivity for cluster of differentiation (CD) markers CD73, CD90, and CD105 and negativity for CD14, CD20, CD34, CD45, and human leukocyte antigen-DR (HLA-DR), or documented in previous publications using previously characterised hPDLSC cultures. Passage numbers between 2 and 9 were accepted, as this range allows adequate cell expansion while maintaining stem/progenitor characteristics for experimental procedures. Studies involving immortalised cell lines, animal-derived cells, or cells obtained from diseased periodontal tissues were excluded.

The interventions included calcium silicate-based endodontic sealers, whether commercially available or experimental, developed specifically for root canal filling and sealing. This category included premixed hydraulic calcium silicate-based sealers (e.g., EndoSequence BC Sealer, TotalFill BC Sealer (FKG Dentaire Sàrl, Le Crêt-du-Locle, Switzerland), AH Plus Bioceramic Sealer (Maruchi, Wonju, Republic of Korea), BioRoot RCS, Bio-C Sealer (Angelus, Londrina, Paraná, Brazil), CeraSeal (Meta Biomed Co., Ltd., Cheongju, Republic of Korea), Well-Root ST (VERICOM Co., Ltd., Chuncheon, Republic of Korea), and NeoSEALER Flo (Avalon Biomed, Houston, Texas, USA)), MTA-containing sealers formulated for obturation (e.g., MTA Fillapex), and clearly identified experimental calcium silicate-based root canal sealers (e.g., C-Root (Beijing C-Root Dental Medical Devices Co., Ltd., Beijing, China)). Experimental sealers were included when they were clearly identified and evaluated as root canal filling or sealing materials because their cellular effects were directly relevant to the mechanistic objectives of this review, irrespective of commercial availability. Studies were excluded if they examined hydraulic calcium silicate-based materials intended primarily for vital pulp therapy, pulp capping, perforation repair, or root-end filling without concurrent assessment as root canal sealers.

The comparators included no intervention (negative control with culture medium only), a positive cytotoxic control (e.g., dimethyl sulfoxide (DMSO)), or alternative sealer types, including epoxy resin-based or silicone-based sealers. The principal focus of the review was on calcium silicate-based sealer arms, while non-calcium silicate materials were considered reference comparators.

The primary outcomes included cell viability and cytotoxicity assessed using MTT, CCK-8, or equivalent metabolic assays, cell migration capacity assessed using wound-healing/scratch assays, and mineralisation potential assessed using Alizarin Red S staining. Secondary outcomes included cell morphology and attachment assessed by scanning electron microscopy or phalloidin staining, osteogenic and cementogenic gene expression assessed by RT-qPCR for markers including RUNX2, ALP, osteocalcin (OCN), CEMP1, and CAP, cytokine production including IL-6, IL-8, and other inflammatory mediators, and physicochemical properties such as pH and calcium or silicon ion release.

Eligible study designs comprised in vitro experimental studies published as peer-reviewed full-text articles. Both parallel-group designs, in which multiple sealers were tested simultaneously on separate cell cultures, and time-series designs, in which a sealer was assessed at multiple time points, were included. Adherence to International Organization for Standardization (ISO) 10993-5 and ISO 10993-12 standards for cytotoxicity testing and sample preparation [19,20], or compliance with the Preferred Reporting Items for Laboratory Studies in Endodontology (PRILE) 2021 guidelines [21], was recorded as an indicator of methodological or reporting quality but was not mandatory for inclusion. Studies using either two-dimensional monolayer cultures or three-dimensional spheroid/scaffold models were eligible. In vivo animal studies, ex vivo human tissue studies without hPDLSC isolation and culture, clinical trials, case reports, case series, narrative reviews, systematic reviews, letters to the editor, editorials, conference abstracts without full-text availability, dissertations, theses, and unpublished data were excluded. Systematic reviews were screened only for potentially relevant additional references. Table 1 summarises the inclusion and exclusion criteria.

**Table 1:** Summary of the inclusion and exclusion criteria. hPDLSCs: Human periodontal ligament stem cells; hPDLCs: Human periodontal ligament cells; MSC: Mesenchymal stem cell; CD: Cluster of differentiation; HLA-DR: Human leukocyte antigen-DR; DPSCs: Dental pulp stem cells; SCAPs: Stem cells from the apical papilla; SHEDs: Stem cells from human exfoliated deciduous teeth; BMMSCs: Bone marrow-derived mesenchymal stem cells; PDL: Periodontal ligament; MTA: Mineral trioxide aggregate; VPT: Vital pulp therapy; CS: Calcium silicate; ALP: Alkaline phosphatase; ISO: International Organization for Standardization.

Criteria	Inclusion	Exclusion
Study design	In vitro experimental studies with controlled conditions, appropriate replication, and statistical analysis	In vivo studies, ex vivo tissue studies, clinical trials, case reports, reviews, letters, editorials, and conference abstracts
Cell population	Primary hPDLSCs or hPDLCs with documented stem/progenitor characteristics from healthy human teeth, characterised by MSC markers (CD73+, CD90+, CD105+, CD14-, CD20-, CD34-, CD45-, HLA-DR-), or previously characterised hPDLSC cultures, at passages 2-9	Other dental stem cells (DPSCs, SCAPs, SHEDs, BMMSCs), PDL fibroblasts without stem cell characterisation, immortalised cell lines, and animal cells
Intervention	Calcium silicate-based root canal sealers (premixed hydraulic sealers, MTA-containing sealers, and clearly identified experimental sealers)	Hydraulic calcium silicate-based cements for VPT/repair only (Biodentine, MTA, iRoot FS, Bio-C Repair); experimental formulations without clear identification or composition
Comparison	Negative control (unconditioned medium), positive cytotoxic or osteogenic control, and/or non-CS comparator sealers	Studies without any control group (unless comparing multiple CS sealers)
Outcomes	At least one relevant cell-based biological outcome, including viability/cytotoxicity, migration, morphology/attachment, mineralisation, osteogenic or cementogenic gene expression, ALP activity, or cytokine production	Studies reporting only physicochemical properties without cell-based biological outcomes
Extraction protocol	Clearly documented extraction or exposure protocol, including material condition, dilution, extraction duration, and exposure method. Compliance with ISO 10993-5, ISO 10993-12, or equivalent protocols was recorded as a methodological quality indicator but was not required for inclusion.	Insufficiently documented extraction or exposure protocols preventing interpretation of the experimental conditions
Publication status	Published peer-reviewed full-text articles	Conference abstracts without full text, dissertations, theses, and unpublished data

Database selection and search strategy

A comprehensive systematic literature search was conducted across four major bibliographic databases selected for their coverage of biomedical and dental research: PubMed/MEDLINE (U.S. National Library of Medicine), Scopus (Elsevier), Web of Science Core Collection (Clarivate Analytics), and CENTRAL. Google Scholar was used as a supplementary search source, with screening limited to the first 200 results ranked by relevance as a pragmatic approach to supplementary searching. The search was conducted on February 1, 2026, with the lower date limit set at January 2016. The search was restricted to English-language publications. Reference lists of relevant systematic reviews were also screened to identify potentially eligible additional studies.

Search Terms and Strategy

The search strategy combined three concept groups using Boolean operators: (1) material terms, (2) cell type terms, and (3) outcome terms. The core search strings used for each database are listed in Table 2.

**Table 2:** Database-specific search strings. MEDLINE: Medical Literature Analysis and Retrieval System Online; MeSH: Medical Subject Headings; PDL: Periodontal ligament; MSC: Mesenchymal stem cell; hPDLSC: Human periodontal ligament stem cell; CENTRAL: Cochrane Central Register of Controlled Trials; TITLE-ABS-KEY: Title, abstract, and keywords; HCSS: Hydraulic calcium silicate-based sealer; CSCS: Calcium silicate-based cement/sealer; MTA: Mineral trioxide aggregate; RUNX2: Runt-related transcription factor 2; ALP: Alkaline phosphatase; COL1A1: Collagen type I alpha 1 chain; CEMP1: Cementum protein 1; hPDLSCs: Human periodontal ligament stem cells; PDLSCs: Periodontal ligament stem cells; hPDLCs: Human periodontal ligament cells; TS: Topic.

Database	Keywords
PubMed/MEDLINE	("Periodontal Ligament"[Mesh] OR "PDL" OR "periodontium") AND ("Stem Cells"[Mesh] OR "stem cell" OR "progenitor cell" OR "mesenchymal stem cell" OR "MSC" OR "hPDLSC" OR "PDL stem cell" OR "periodontal stem cell") AND ("Root Canal Filling Materials"[Mesh] OR "Dental Cements"[Mesh] OR "endodontic sealer" OR "root canal sealer" OR "obturation material" OR "bioceramic" OR "calcium silicate" OR "Calcium Compounds"[Mesh] OR "Silicates"[Mesh] OR "tricalcium silicate" OR "dicalcium silicate" OR "hydraulic calcium silicate" OR "CSCS") AND ("Biocompatible Materials"[Mesh] OR "cytocompatible" OR "cell viability" OR "cell proliferation" OR "cell migration" OR "Cell Differentiation"[Mesh] OR "Osteogenesis"[Mesh] OR "differentiation" OR "osteogenesis" OR "cementogenesis" OR "bioactivity" OR "Gene Expression"[Mesh] OR "RUNX2" OR "ALP" OR "alkaline phosphatase") NOT ("animals"[Mesh] NOT "humans"[Mesh])
Scopus	(TITLE-ABS-KEY("periodontal ligament" OR pdl OR periodontium) AND TITLE-ABS-KEY("stem cell*" OR "progenitor cell*" OR "mesenchymal stem cell*" OR msc OR hpdlsc* OR "pdl stem cell*")) AND (TITLE-ABS-KEY("endodontic sealer*" OR "root canal sealer*" OR "obturation material*" OR bioceramic* OR "calcium silicate*" OR "tricalcium silicate*" OR "dicalcium silicate*" OR "hydraulic calcium silicate*" OR hcss OR cscs) OR TITLE-ABS-KEY(endosequence OR totalfill OR bioroot OR "bio-c sealer" OR "ah plus bioceramic" OR ceraseal OR endoseal OR wellroot OR neosealer OR "mta fillapex" OR "iroot sp" OR "guttaflow bioseal")) AND TITLE-ABS-KEY(biocompatib* OR cytotoxic* OR cytocompat* OR "cell viab*" OR "cell prolif*" OR "cell migrat*" OR differentiat* OR osteogen* OR cementogen* OR mineraliz* OR bioactiv* OR regenerat* OR runx2 OR alp OR "alkaline phosphatase" OR col1a1 OR cemp1 OR osteocalcin)
Web of Science	TS=("calcium silicate" OR "bioceramic sealer" OR "hydraulic sealer" OR "EndoSequence" OR "BioRoot" OR "TotalFill" OR "CeraSeal" OR "BC Sealer" OR "MTA sealer") AND TS=("periodontal ligament stem cells" OR "hPDLSCs" OR "PDLSCs" OR "periodontal ligament cells" OR "hPDLCs") AND TS=("cytotoxicity" OR "biocompatibility" OR "cell viability" OR "cytocompatibility" OR "bioactivity" OR "osteogenic" OR "cementogenic")
Cochrane Central Register of Controlled Trials (CENTRAL)	("periodontal ligament" OR PDL OR periodontium) AND ("stem cell" OR "mesenchymal stem cell" OR MSC OR hPDLSC OR "PDL stem cell") AND ("endodontic sealer" OR "root canal sealer" OR "obturation material" OR bioceramic OR "calcium silicate" OR "tricalcium silicate" OR "hydraulic calcium silicate") AND (biocompatible OR cytotoxicity OR cytocompatibility OR "cell viability" OR differentiation OR osteogenesis OR cementogenesis OR mineralization)
Google Scholar	"periodontal ligament stem cells" ("calcium silicate" OR bioceramic) sealer (biocompatibility OR bioactivity OR differentiation)

Search Documentation

The complete search strategies for each database, including platform-specific syntax, filters applied, and results obtained, were documented in a supplementary search log. The search results were exported to reference management software (Zotero, Corporation for Digital Scholarship, Vienna, Virginia, USA) for duplicate removal, and the accuracy of the deduplication process was confirmed by manual verification. After duplicate removal and Zotero-based automation-assisted filtering, the remaining records were exported to Covidence systematic review software (Veritas Health Innovation, Melbourne, Australia) for reviewer screening, full-text assessment, and final study selection.

Study Selection Process

After removal of 287 duplicate records in Zotero and manual verification, 26 records were marked as ineligible by automation before screening, leaving 578 records for title and abstract screening. During screening, 335 records were excluded using a Zotero-based automation-assisted filtering procedure, while two reviewers (AV, IN) independently assessed the remaining records against the eligibility criteria. A two-stage screening protocol was employed. Stage 1 involved title screening to exclude clearly irrelevant studies based on material type (non-endodontic or non-calcium silicate), study type (reviews or case reports), or population (animal studies or non-periodontal cells). Stage 2 involved abstract screening using the full eligibility criteria. The reviewers excluded an additional 175 records, leaving 68 reports sought for retrieval. Disagreements during screening were resolved through discussion, and the final set of included studies was determined by consensus after full-text assessment. The selection process was reported in accordance with PRISMA 2020 [17], and the complete process is illustrated in the PRISMA flowchart (Figure 1).

**Figure 1:**
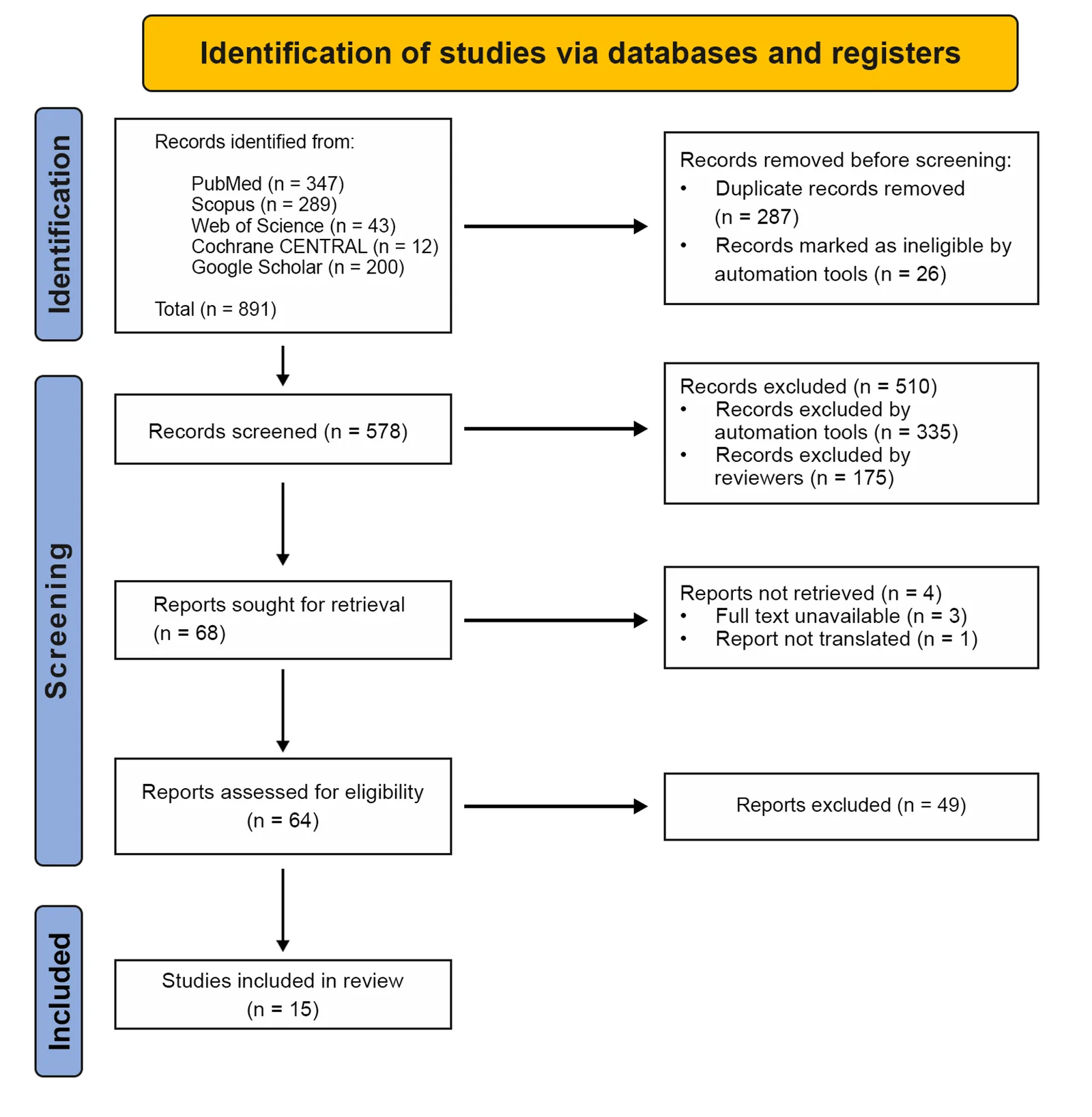
PRISMA flowchart. PRISMA: Preferred Reporting Items for Systematic Reviews and Meta-Analyses; CENTRAL: Cochrane Central Register of Controlled Trials.

Data Collection and Extraction

A standardised data extraction form was specifically designed for in vitro endodontic biocompatibility studies. Two reviewers independently extracted data from all included studies using this standardised form. Discrepancies in the extracted data between reviewers were resolved through re-examination of the source publication, and any disagreements that persisted after re-examination were adjudicated by a third reviewer. All extracted data were entered into a master database with version control and change tracking enabled. Logical consistency checks were performed to identify data entry errors (e.g., sample sizes not matching across outcomes within the same study and potential statistical anomalies in the reported data). Regular backups were maintained throughout the review process, and the final database was archived after the completion of data synthesis.

Assessment of methodological quality

Since there is no specific tool to rate the risk of bias for in vitro studies, the NTP-OHAT Risk of Bias Rating Tool for Human and Animal Studies was used and adapted for this systematic review [22]. The tool has previously been used in systematic reviews of in vitro toxicological studies and was used to assess selected aspects of methodological quality across seven domains.

Domain 1 (selection bias) assessed the extent of reporting of cell donor characteristics (age, sex, health status, and tooth type), whether group allocation was adequately reported, and whether systematic differences between experimental groups were likely. Domain 2 (performance bias) assessed whether experimental procedures and material exposures were standardised across groups. Domain 3 (blinding of experimental personnel) evaluated whether investigators conducting the experimental procedures were blinded to group allocation. Domain 4 (detection bias/outcome assessment) assessed whether outcome assessment was performed without knowledge of the experimental groups or whether objective or automated assessment methods were used. Domain 5 (attrition bias) assessed incomplete outcome data or unexplained loss of experimental samples. Domain 6 (selective reporting bias) evaluated whether all prespecified or expected outcomes were reported. Domain 7 (other sources of bias) considered additional threats to validity, including inappropriate statistical methods, pseudoreplication, inadequate independent replication, undeclared conflicts of interest, and potentially influential funding sources. This domain received higher risk ratings for studies that were directly funded by the manufacturer and lacked independent oversight.

Risk of bias rating process

Two reviewers independently assessed each included study across all seven OHAT domains, assigning adapted ratings of low, unclear, or high risk based on predefined criteria for in vitro research. Disagreements were resolved through discussion. Overall risk-of-bias judgements were categorised as low, unclear, or high based on the pattern of domain-level ratings across the seven domains. Risk-of-bias assessments were visualised using traffic-light plots generated in R software (R Foundation for Statistical Computing, Vienna, Austria) with the robvis package [23].

The risk-of-bias evaluation across the seven OHAT domains for the 15 included studies is summarised in Figure 2. Most studies demonstrated a low overall risk of bias, while one study was rated as having an unclear overall risk of bias. Unclear risk was observed most frequently in Domain 4 (detection bias/outcome assessment) and, to a lesser extent, in Domain 3 (blinding of experimental personnel). No study was rated as having a high overall risk of bias. Incomplete reporting of outcome-assessor blinding may reduce confidence in outcomes requiring subjective or semi-quantitative assessment, although most studies used standardised laboratory procedures and objective quantitative assays.

**Figure 2:**
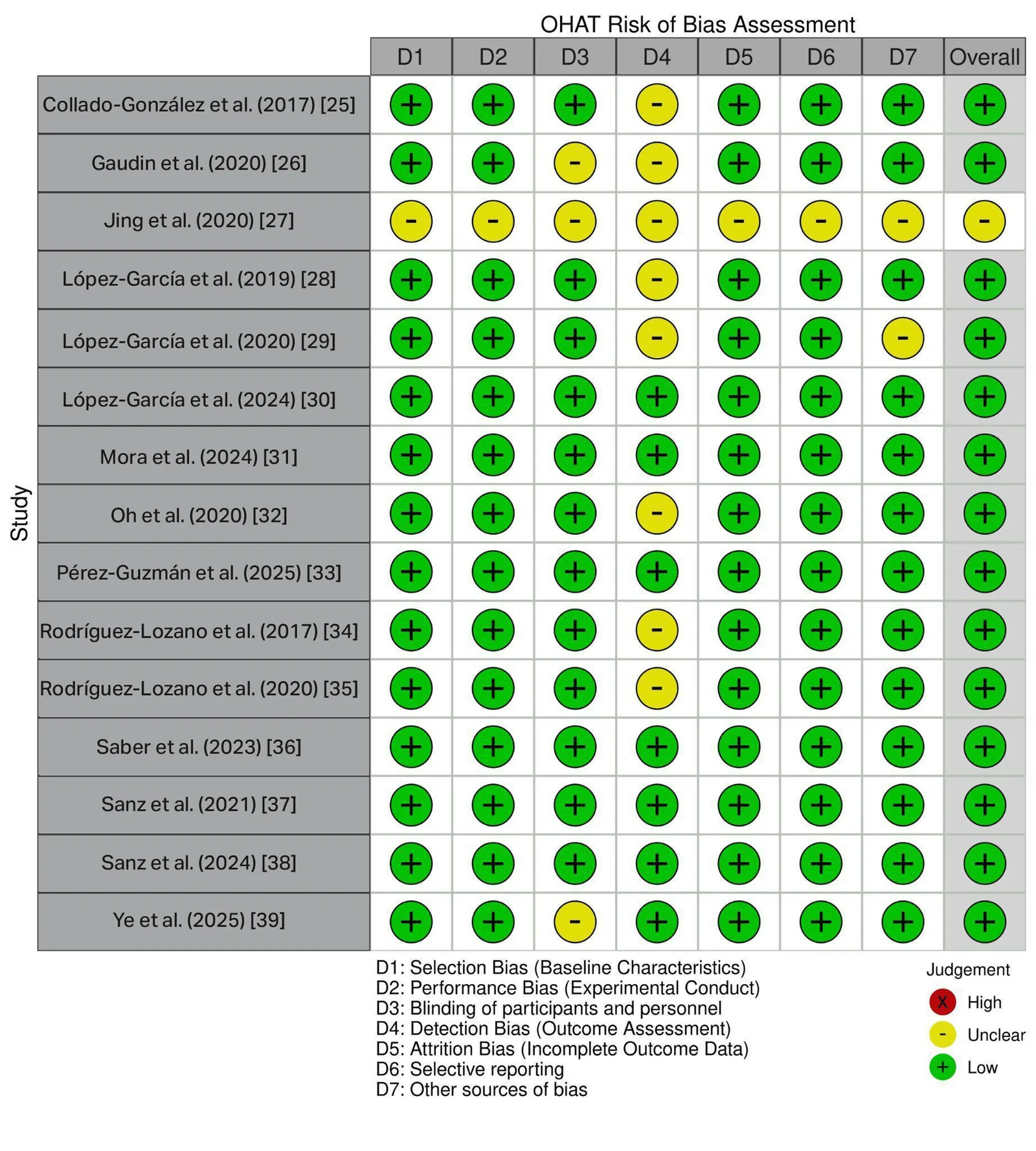
Risk-of-bias assessment of the included studies. Green indicates low risk of bias, yellow indicates unclear risk of bias, and red indicates high risk of bias.

Although the core variables were conceptually similar, substantial heterogeneity in root canal sealers, cell sources, extract preparation (fresh versus set materials, extraction durations, and dilution series), assay protocols, and effect measures precluded a statistically valid meta-analysis. Consequently, a structured narrative synthesis was conducted following published guidance for the narrative synthesis of quantitative data [24] to summarise and explore patterns across studies. Differences in outcomes across studies therefore cannot be attributed solely to sealer formulation, and direct cross-study comparisons should be interpreted cautiously.

Results

Database Search Yield

A total of 891 records were identified from PubMed/MEDLINE (n = 347), Scopus (n = 289), Web of Science Core Collection (n = 43), CENTRAL (n = 12), and Google Scholar (n = 200). After removal of 287 duplicate records and 26 records marked as ineligible by automation before screening (n = 313), 578 records remained for title and abstract screening. Title and abstract screening resulted in the exclusion of 510 records, leaving 68 reports sought for retrieval. Four reports were not retrieved, and 64 reports were assessed for eligibility, of which 49 were excluded after full-text assessment. The final qualitative synthesis included 15 studies, as shown in the PRISMA flowchart (Figure 1) [17], and the findings of each study are summarised in Table 3.

**Table 3:** Cross-study comparison of in vitro hPDLSC responses to calcium silicate-based endodontic sealers. NR indicates that the study did not assess this outcome. Comparator groups, exposure periods, and statistical significance are reported where available. 2D: Two-dimensional; 3D: Three-dimensional; AD-S: ADSeal; AHP: AH Plus; AHP BC/AHPbc/AHPbcs: AH Plus Bioceramic Sealer; ALP: Alkaline phosphatase; ARS: Alizarin Red S; BCI: Bio-C Sealer ION+; BCHiF/ES: EndoSequence BC Sealer HiFlow; BCS/ES BC: EndoSequence BC Sealer; BrF: BioRoot Flow; BSP: Bone sialoprotein; CAP: Cementum attachment protein; CEMP1: Cementum protein 1; COL1A1: Collagen type I alpha 1 chain; CP23: Cementum protein 23; CS: CeraSeal; ECM: Extracellular matrix; GF: GuttaFlow 2; hPDLSCs/PDLSCs: Human periodontal ligament stem cells; mRNA: Messenger RNA; MTA: Mineral trioxide aggregate; MTT: 3-(4,5-dimethylthiazol-2-yl)-2,5-diphenyltetrazolium bromide; NCS: Nano-ceramic sealer; Neo/NS Flo: NeoSEALER Flo; NS EZ Flo: NeoSEALER EZFlo; NR: Not reported; OCN: Osteocalcin; OPG: Osteoprotegerin; RCS: Root canal sealer; RT-qPCR: Reverse transcription quantitative polymerase chain reaction; RUNX2: Runt-related transcription factor 2; TFbc: TotalFill BC Sealer; VDW.1: VDW.1Seal; WR-ST: Well-Root ST.

Author (Year)	Region	Main Aim	Key Materials / Sealers	Cell Viability / Proliferation	Cell Migration / Scratch Assay	Mineralisation (Alizarin Red)	Osteogenic / Cementogenic Gene Expression	Main Conclusion
Collado-González M et al. (2017) [25]	Spain	Evaluate the biocompatibility of three calcium silicate-based sealers on hPDLSCs	BioRoot BC Sealer, EndoSeal MTA, Nano-ceramic Sealer (NCS)	At 72 h, BioRoot showed high proliferation, NCS moderate proliferation, and EndoSeal low proliferation (p < 0.05).	Scratch assay: BioRoot showed higher migration than the other tested sealers (p < 0.05).	NR	NR	BioRoot and NCS were more cytocompatible than EndoSeal MTA.
Gaudin A et al. (2020) [26]	France/USA	Evaluate the effects of different calcium silicate-based sealers on the viability and cytokine production of PDLSCs	BioRoot RCS, ProRoot ES, MTA Fillapex (vs AH Plus)	BioRoot RCS maintained favourable PDLSC viability, whereas MTA Fillapex was strongly cytotoxic even at low extract dilutions; ProRoot ES showed no adverse effect on viability.	NR	NR	NR	BioRoot RCS and ProRoot ES showed favourable cytocompatibility, whereas MTA Fillapex was strongly cytotoxic in 2D culture; BioRoot RCS and ProRoot ES also promoted an anti-inflammatory cytokine response.
Jing Y et al. (2020) [27]	China & Hong Kong	Assess the cytocompatibility, osteogenic potential, and inflammatory response of an experimental calcium silicate-based cement in hPDLSCs	BioRoot RCS (tricalcium silicate), C-Root (experimental calcium silicate) vs AH Plus (epoxy)	BioRoot and C-Root increased proliferation over time; AH Plus showed the lowest proliferation.	NR	BioRoot and AH Plus produced more mineralised nodules than the control.	C-Root showed higher ALP, OCN, and RUNX2 mRNA expression than AH Plus at day 7.	C-Root and BioRoot are cytocompatible; C-Root has greater osteogenic potential than AH Plus.
López-García S et al. (2019) [28]	Spain	To investigate the cytocompatibility and mineralisation potential of two premixed hydraulic endodontic sealers compared with an epoxy resin-based root canal sealer	Bio-C Sealer, TotalFill BC Sealer vs AH Plus	AH Plus reduced cell viability and cell migration, whereas increased cell viability and cell migration were observed with Bio-C Sealer and TotalFill BC Sealer (p < 0.05).	Scratch assay: Bio-C and TotalFill increased migration.	At the end of 21 days, only Bio-C Sealer and TotalFill BC Sealer supported matrix mineralisation (p < 0.05).	NR (focus on mineralisation and morphology)	Bio-C and TotalFill were more cytocompatible than AH Plus in terms of cell viability, migration, cell morphology, cell attachment, and mineralisation.
López-García S et al. (2020) [29]	Spain	Compare the biological properties of EndoSequence BC Sealer, CeraSeal, and EndoSeal MTA	EndoSequence BC Sealer, CeraSeal, EndoSeal MTA	EndoSequence BC Sealer and CeraSeal showed higher viability than EndoSeal MTA, which significantly reduced viability.	EndoSeal reduced migration; EndoSequence and CeraSeal supported migration.	EndoSequence and CeraSeal showed greater mineralisation than EndoSeal.	CeraSeal and EndoSequence induced ALP, CAP, and CEMP1 expression and a significantly higher mineralisation capacity than EndoSeal (p < 0.001).	EndoSequence BC Sealer and CeraSeal showed superior bioactivity and cytocompatibility compared with EndoSeal.
López-García S et al. (2024) [30]	Spain	Assess the effects of premixed ceramic sealers on viability and osteo-/cementogenic differentiation of hPDLSCs	TotalFill BC Sealer (TFbc), AH Plus Bioceramic Sealer (AHPbc), and NeoSEALER Flo (Neo)	MTT assays revealed a significant reduction in mitochondrial activity after exposure to Neo extracts at all dilutions and times evaluated compared with the negative control (p < 0.001).	Neo showed limited migration (p < 0.001); TFbc showed better migration and adhesion.	All increased mineralisation compared with the control; TFbc showed the most pronounced calcium deposition.	All induced CEMP1, ALP, and COL1A1; the strongest early overexpression was observed with TFbc.	TFbc most strongly promoted osteo-/cementogenic gene expression and ECM mineralisation; responses with Neo and AHPbc were less pronounced.
Mora A et al. (2024) [31]	Spain	Assess the biocompatibility, bioactivity, and immunomodulatory effects of CeraSeal (CS), TotalFill BC Sealer (TFbc), and Well-Root ST (WR-ST) on hPDLSCs	CeraSeal, TotalFill BC Sealer, Well-Root ST	All three were similar to the control at 1:2-1:4 dilutions; undiluted extracts reduced metabolic activity at 48-72 h.	Wound-healing assay: all sealers allowed migration comparable to the control.	CS and TFbc > negative and positive controls; CS > TFbc; WR-ST ≈ controls.	CS and TFbc upregulated BSP, CEMP1, CAP, and RUNX2; the strongest response was observed with CS.	All sealers were cytocompatible; CS was the strongest inducer of osteo-/cementogenic differentiation and mineralisation.
Oh H et al. (2020) [32]	Korea	Compare the biocompatibility and osteogenic potential of CeraSeal and EndoSeal TCS with AH Plus	CeraSeal, EndoSeal TCS vs AH Plus	AH Plus showed the lowest viability; CeraSeal showed the highest viability.	NR	ALP and ARS staining: CeraSeal and EndoSeal TCS were similar to the osteogenic positive control; AH Plus showed less staining.	RT-qPCR: ALP, RUNX2, and OCN expression was higher for EndoSeal TCS and CeraSeal than for AH Plus.	Calcium silicate-based sealers were more biocompatible and osteogenic than epoxy resin-based AH Plus.
Pérez-Guzmán N et al. (2026) [33]	Spain	Examine the biocompatibility, regenerative, and immunomodulatory effects of TotalFill BC, NeoSEALER Flo, and NeoSEALER EZ Flo	TotalFill BC Sealer, NeoSEALER Flo, NeoSEALER EZ Flo	Bioceramic sealers increased viability over time; all demonstrated adequate cytocompatibility.	All tested sealers supported cell migration, with the most favourable migration response observed with TFbc.	After 21 days, ARS staining revealed a significant enhancement in mineral deposition in the NS Flo group compared with the TFbc, NS EZ Flo, and positive control groups.	NS Flo showed the most pronounced upregulation at day 7 of ALP, CEMP1, CAP, and RUNX2 compared with the control and OsteoDiff groups.	All bioceramic sealers were cytocompatible; NeoSEALER Flo and NeoSEALER EZ Flo showed strong differentiation and mineralisation responses, and all demonstrated anti-inflammatory effects.
Rodríguez-Lozano FJ et al. (2017) [34]	Spain	Compare the cytocompatibility of MTA Fillapex and TotalFill BC Sealer with AH Plus	TotalFill BC Sealer, MTA Fillapex, AH Plus	TotalFill BC Sealer increased proliferation compared with AH Plus and MTA Fillapex and showed lower cytotoxicity.	TotalFill promoted greater migration in the scratch assay than the other sealers in a concentration-dependent manner.	NR	NR	TotalFill BC Sealer was more cytocompatible and pro-migratory than AH Plus and MTA Fillapex.
Rodríguez-Lozano FJ et al. (2020) [35]	Spain	Evaluate, in a laboratory setting, the effects of EndoSequence BC Sealer HiFlow on hPDLSCs	EndoSequence BC Sealer HiFlow (BCHiF), EndoSequence BC Sealer (BCS) vs AH Plus (AHP)	The cell viability assay revealed no relevant differences between BCS and BCHiF compared with the control group (p > 0.05).	Both BCS and BCHiF showed similar rates of cell migration to the control group at 24 and 48 h.	The Alizarin Red assay revealed a significant difference between BCS and the control group (p < 0.001), as well as between BCHiF and the control group (p < 0.001).	BCS and BCHiF promoted overexpression of osteo-/cementogenic genes (ALP, CEMP1, and RUNX2).	EndoSequence BC Sealer HiFlow possesses suitable biological properties for use as a root canal filling material.
Saber S et al. (2023) [36]	Egypt	Compare the cytocompatibility and osteogenic potential of VDW.1Seal (VDW.1), EndoSequence BC Sealer HiFlow (ES), GuttaFlow 2 (GF), and ADSeal (AD-S) on hPDLSCs	VDW.1Seal, EndoSequence BC Sealer HiFlow (ES), GuttaFlow 2, ADSeal	GuttaFlow 2 generally showed the highest cell viability among the tested sealers.	GuttaFlow 2 showed the lowest open wound area, indicating the greatest wound closure.	GuttaFlow 2 and ADSeal showed the highest calcium nodule deposition by ARS.	RUNX2 and OCN expression was similar for all sealers; OPG expression was higher for VDW.1 and GuttaFlow 2.	GuttaFlow 2 and VDW.1Seal showed the most favourable overall behaviour towards host stem cells.
Sanz JL et al. (2021) [37]	Spain	Assess the microstructure, ion release, cytocompatibility, and mineralisation of Bio-C Sealer ION+ (BCI) and EndoSequence BC Sealer HiFlow compared with AH Plus	Bio-C Sealer ION+ (BCI), EndoSequence BC Sealer HiFlow (BCHiF), AH Plus	BCI and BCHiF showed adequate cytocompatibility; AH Plus was less favourable.	BCI and BCHiF supported migration.	BCI and BCHiF showed greater mineralised nodule formation than the controls (p < 0.001).	The BCHiF group showed upregulation of CAP (p < 0.01), CEMP1, ALP, and RUNX2 (p < 0.001) compared with the negative control, while the BCI group showed upregulation of CEMP1 (p < 0.01), CAP, and RUNX2 (p < 0.001).	BCI and BCHiF showed high calcium ion release, good cytocompatibility, and strong osteo-/cementogenic differentiation.
Sanz JL et al. (2024) [38]	Spain	Evaluate the cytocompatibility, bioactivity, and anti-inflammatory potential of BioRoot Flow (BrF) and AH Plus Bioceramic Sealer (AHPbcs) compared with AH Plus	BioRoot Flow (BrF), AH Plus Bioceramic Sealer (AHPbcs) vs AH Plus	BrF and AHPbcs showed positive metabolic activity and attachment; AH Plus showed less favourable responses.	BrF and AHPbcs increased migration and proliferation in the wound-healing assay; AH Plus impaired these responses.	BrF > AHPbcs > controls; both showed greater ARS mineralisation than AH Plus.	BrF upregulated CAP and BSP (osteo-/cementogenic markers) compared with both controls; CEMP1 expression in both groups was similar to that of the control groups at 7 days but significantly declined after 21 days of culture (p < 0.01 for AHPbcs; p < 0.001 for BrF).	BrF and AHPbcs were cytocompatible and immunomodulatory; BrF particularly enhanced mineralisation and differentiation.
Ye Y et al. (2025) [39]	Australia	Compare the cytotoxicity, biocompatibility, and bioactivity of EndoSequence BC Sealer (ES BC) and AH Plus Bioceramic Sealer (AHP BC) with AH Plus	EndoSequence BC Sealer (ES BC) and AH Plus Bioceramic Sealer (AHP BC) vs AH Plus	ES BC and AHP BC showed higher viability/proliferation than conventional AH Plus.	ES BC and AHP BC enhanced migration compared with the control; AH Plus showed a less favourable response.	NR	Set ES BC markedly upregulated ALP, RUNX2, and COL1A1 more than cementogenic markers (CAP, CP23, and CEMP1).	ES BC and AHP BC showed superior biocompatibility and regenerative, mainly osteogenic, capacity compared with AH Plus.

Risk of Bias Assessment

The traffic-light diagram summarises the OHAT risk-of-bias assessment for the included in vitro studies evaluating calcium silicate-based sealers. Green circles indicate low risk of bias, yellow circles indicate unclear risk, and red circles indicate high risk of bias. Across most domains, the included studies were predominantly rated as having a low risk of bias. Unclear ratings were most frequent in Domain 4 (D4; detection bias/outcome assessment), affecting eight studies: Collado-González M et al. (2017) [25], Gaudin A et al. (2020) [26], Jing Y et al. (2020) [27], López-García S et al. (2019) [28], López-García S et al. (2020) [29], Oh H et al. (2020) [32], Rodríguez-Lozano FJ et al. (2017) [34], and Rodríguez-Lozano FJ et al. (2020) [35]. Domain 3 (D3; blinding of experimental personnel) was rated as unclear in Gaudin A et al. (2020) [26], Jing Y et al. (2020) [27], and Ye Y et al. (2025) [39]. Unclear ratings in these domains primarily reflected insufficient reporting of blinding procedures, which may reduce confidence in outcomes involving subjective or semi-quantitative assessment.

Jing Y et al. (2020) [27] received unclear ratings across all seven domains and was therefore rated as having an unclear overall risk of bias. López-García S et al. (2020) [29] additionally received an unclear rating in D7 (other sources of bias), while retaining a low overall risk of bias. All remaining studies were rated as having a low overall risk of bias. No high-risk ratings were identified in any individual domain or in the overall assessments. Overall, the findings indicate generally low methodological risk across the included studies, although incomplete reporting of blinding and outcome-assessment procedures highlights an area requiring greater standardisation in future in vitro research.

Qualitative Synthesis

This systematic review compiled evidence from 15 in vitro studies investigating the biological interactions between calcium silicate-based endodontic sealers and hPDLSCs and several crucial cellular mechanisms relevant to regenerative endodontics. The analysed sealers were classified based on their chemical composition (Table 4), which may affect their biological properties.

**Table 4:** Summary of all root canal sealers evaluated across the included in vitro studies. AHP: AH Plus; AHP BC/AHPbc/AHPbcs: AH Plus Bioceramic Sealer; BCI: Bio-C Sealer ION+; BCS/ES BC: EndoSequence BC Sealer; BCHiF/ES: EndoSequence BC Sealer HiFlow; BrF: BioRoot Flow; MTA: Mineral trioxide aggregate; NCS: Nano-ceramic sealer; Neo/NS Flo: NeoSEALER Flo; NS EZ Flo: NeoSEALER EZFlo; RCS: Root canal sealer; TCS: Tricalcium silicate; TFbc: TotalFill BC Sealer; VDW.1: VDW.1Seal; WR-ST: Well-Root ST.

Material	Category	Composition	Studies
BioRoot RCS / BioRoot BC Sealer (Septodont, Saint-Maur-des-Fossés, France)	Calcium silicate-based (tricalcium silicate) sealer	Powder: tricalcium silicate, zirconium oxide, and povidone; liquid: aqueous solution of calcium chloride and polycarboxylate	Jing Y et al. (2020) [27]; Collado-González M et al. (2017) [25]; Gaudin A et al. (2020) [26]
C-Root	Experimental calcium silicate-based root canal filling material	Powder: calcium silicate compound, strontium silicate, calcium dihydrogen phosphate, potassium dihydrogen phosphate, magnesium oxide, and zirconium dioxide; liquid: polyethylene glycol, water-free liquid	Jing Y et al. (2020) [27]
MTA Fillapex (Angelus, Londrina, Paraná, Brazil)	Calcium silicate-containing sealer	Paste A: salicylate resin, bismuth trioxide, and fumed silica; Paste B: fumed silica, titanium dioxide, mineral trioxide aggregate, and base resin	Rodríguez-Lozano FJ et al. (2017) [34]; Gaudin A et al. (2020) [26]
TotalFill BC Sealer (TFbc)	Premixed calcium silicate-based/bioceramic sealer	Zirconium oxide, tricalcium silicate, dicalcium silicate, and calcium hydroxide	Rodríguez-Lozano FJ et al. (2017) [34]; López-García S et al. (2019) [28]; Mora A et al. (2024) [31]; Pérez-Guzmán N et al. (2026) [33]; López-García S et al. (2024) [30]
Nano-ceramic Sealer (NCS)	Calcium silicate-based nano-ceramic sealer	Calcium silicates, zirconium oxide, filler, and thickening agent	Collado-González M et al. (2017) [25]
EndoSeal MTA / EndoSeal	Calcium silicate-based/MTA-type sealer	Calcium silicates, calcium aluminates, calcium sulphate, radiopacifier, and thickening agent	Collado-González M et al. (2017) [25]; López-García S et al. (2020) [29]
Bio-C Sealer	Premixed calcium silicate-based sealer	Tricalcium silicate, dicalcium silicate, tricalcium aluminate, calcium oxide, zirconium oxide, silicon dioxide, polyethylene glycol, and iron oxide	López-García S et al. (2019) [28]
Bio-C Sealer ION+ (BCI)	Premixed calcium silicate-based sealer	Calcium silicate, magnesium silicate, polyethylene glycol, zirconium oxide, silicon dioxide nanoparticles, potassium sulphate, and calcium sulphate hemihydrate	Sanz JL et al. (2021) [37]
EndoSequence BC Sealer (ES BC) (Brasseler USA, Savannah, Georgia, USA)	Premixed calcium silicate-based sealer	Zirconium oxide, calcium silicates, calcium phosphate monobasic, calcium hydroxide, filler, and thickening agents	López-García S et al. (2020) [29]; Rodríguez-Lozano FJ et al. (2020) [35]; Ye Y et al. (2025) [39]
EndoSequence BC Sealer HiFlow (ES / BCHiF)	Warm vertical compaction/HiFlow variant of BC Sealer	Zirconium oxide, tricalcium silicate, dicalcium silicate, calcium hydroxide, and fillers	Rodríguez-Lozano FJ et al. (2020) [35]; Saber S et al. (2023) [36]; Sanz JL et al. (2021) [37]
CeraSeal	Calcium silicate-based sealer	Calcium silicates, zirconium oxide, and thickening agent	Mora A et al. (2024) [31]; Oh H et al. (2020) [32]; López-García S et al. (2020) [29]
EndoSeal TCS	Calcium silicate-based sealer	Tricalcium silicate, phyllosilicate mineral, zirconium oxide, and dimethyl sulfoxide	Oh H et al. (2020) [32]
AH Plus Bioceramic Sealer (AHP BC / AHPbcs / AHPbc)	Bioceramic/calcium silicate-containing sealer	Zirconium dioxide, tricalcium silicate, dimethyl sulfoxide, lithium carbonate, and thickening agent	Ye Y et al. (2025) [39]; Sanz JL et al. (2024) [38]; López-García S et al. (2024) [30]
BioRoot Flow (BrF)	Premixed calcium silicate-based sealer	Tricalcium silicate, propylene glycol, povidone, calcium carbonate, aerosil, zirconium oxide, acrylamide/sodium acryloyldimethyltaurate copolymer, isohexadecane, and polysorbate	Sanz JL et al. (2024) [38]
Well-Root ST (WR-ST)	Calcium silicate-based sealer	Calcium aluminosilicate compound, zirconium oxide, filler, and thickening agent	Mora A et al. (2024) [31]
NeoSEALER Flo (Neo / NS Flo)	Premixed bioceramic sealer	Tantalite, tricalcium silicate, calcium aluminate, dicalcium silicate, grossite, tricalcium aluminate, and calcium sulphate	López-García S et al. (2024) [30]; Pérez-Guzmán N et al. (2026) [33]
NeoSEALER EZFlo (NS EZ Flo)	Premixed bioceramic root canal sealer	Tantalite, tricalcium silicate, calcium aluminate, dicalcium silicate, grossite, tricalcium aluminate, and calcium sulphate	Pérez-Guzmán N et al. (2026) [33]
ProRoot ES	Bioceramic root canal sealer	Powder: calcium sulphate, dicalcium silicate, tricalcium silicate, bismuth oxide, and a small amount of tricalcium aluminate; liquid: viscous aqueous solution of a water-soluble polymer	Gaudin A et al. (2020) [26]
VDW.1Seal (VDW.1)	Bioceramic root canal sealer	Zirconium dioxide, tricalcium silicate, dimethyl sulfoxide, lithium carbonate, and thickening agents	Saber S et al. (2023) [36]

Although conceptually similar outcomes were assessed (cell viability/proliferation, migration/wound closure, mineralisation using Alizarin Red S staining, and osteo-/cementogenic gene expression), substantial methodological heterogeneity precluded quantitative meta-analysis. Differences included cell sources and passage numbers, extract preparation (fresh versus set materials and surface area-to-volume ratios), dilution series, exposure durations, and effect metric scales (raw optical density versus normalised percentages). A structured narrative synthesis approach was therefore employed, following established guidance for quantitative data where pooling is infeasible [24], to identify consistent patterns, explore heterogeneity, and contextualise findings within the broader literature on sealer bioactivity. Studies were grouped by outcome category, and conflicting findings were interpreted in relation to differences in assay methods and exposure conditions.

Cell Viability/Proliferation

Evaluation of cell viability and proliferation using MTT and CCK-8 assays revealed distinct outcomes among the sealer types. All 15 studies contributed quantitative data, primarily from MTT (13 studies: Gaudin A et al. (2020) [26], Collado-González M et al. (2017) [25], López-García S et al. (2019) [28], López-García S et al. (2020) [29], López-García S et al. (2024) [30], Rodríguez-Lozano FJ et al. (2017) [34], Rodríguez-Lozano FJ et al. (2020) [35], Mora A et al. (2024) [31], Ye Y et al. (2025) [39], Sanz JL et al. (2021) [37], Sanz JL et al. (2024) [38], Saber S et al. (2023) [36], and Pérez-Guzmán N et al. (2026) [33]) or CCK-8 (two studies: Jing Y et al. (2020) [27] and Oh H et al. (2020) [32]) assays at 24-72 hours. Findings were largely consistent within sealer classes but mixed across all studies due to protocol variations.

Premixed calcium silicate (CS) sealers, particularly EndoSequence BC/TotalFill BC, Bio-C Sealer ION+, and CeraSeal, demonstrated viability comparable to or exceeding negative controls at dilutions ≥1:4 using set material extracts, outperforming epoxy-based AH Plus. TotalFill BC Sealer (TFbc) demonstrated high biocompatibility and was particularly effective in promoting substrate attachment and spreading of hPDLSCs, as reported by López-García S et al. (2019) [28], Pérez-Guzmán N et al. (2026) [33], and Rodríguez-Lozano FJ et al. (2017) [34]. BioRoot RCS and BioRoot Flow consistently showed time-dependent increases in cell proliferation. BioRoot RCS demonstrated the highest proliferation rates from days 1 to 5 in certain models, as reported by Sanz JL et al. (2024) [38], Jing Y et al. (2020) [27], and Gaudin A et al. (2020) [26]. C-Root was shown to have cytocompatibility comparable to BioRoot RCS. It supported significant cell proliferation compared with AH Plus (P < 0.05), as reported by Jing Y et al. (2020) [27].

Cell Migration/Wound Closure (Scratch Assay)

Twelve of the 15 studies analysed cell migration or wound healing. EndoSequence BC HiFlow and TotalFill BC generally supported migration rates equivalent to or greater than those of the controls, whereas epoxy sealers exhibited delayed wound closure in direct comparisons, as reported by Rodríguez-Lozano FJ et al. (2017) [34], Sanz JL et al. (2021) [37], and Ye Y et al. (2025) [39]. NeoSEALER Flo showed comparatively limited migration in one study, as reported by López-García S et al. (2024) [30]. Overall, migration outcomes varied across studies and with extract concentration.

Mineralisation (Alizarin Red Staining)

Of the 11 studies, three assessed mineralisation at 14 days, as reported by Jing Y et al. (2020) [27], Oh H et al. (2020) [32], and Saber S et al. (2023) [36], and eight assessed mineralised-nodule formation at 21 days, as reported by López-García S et al. (2019) [28], López-García S et al. (2020) [29], López-García S et al. (2024) [30], Sanz JL et al. (2021) [37], Sanz JL et al. (2024) [38], Rodríguez-Lozano FJ et al. (2020) [35], Mora A et al. (2024) [31], and Pérez-Guzmán N et al. (2026) [33]. Calcium silicate-based sealers generally induced greater calcium deposition than the controls, with EndoSequence/TotalFill BC variants showing the most pronounced effects; epoxy sealers generally demonstrated lower mineralisation in direct comparisons, as reported by López-García S et al. (2019) [28], López-García S et al. (2020) [29], and López-García S et al. (2024) [30]. BioRoot Flow (BrF) produced significantly greater calcified nodule formation than AH Plus Bioceramic Sealer, AH Plus, and the positive control (OsteoDiff) groups, as reported by Sanz JL et al. (2024) [38].

Gene Expression of Osteogenic and Cementogenic Markers

Eleven studies quantified osteogenic and cementogenic markers, including ALP, RUNX2, CEMP1, and CAP, using RT-qPCR at intervals ranging from 3 to 21 days [27,29,30,31,32,33,35,36,37,38,39]. EndoSequence/TotalFill BC variants upregulated early osteogenic genes (ALP and RUNX2) and cementogenic markers (CEMP1 and CAP) in most experiments, exceeding epoxy sealer baselines. Calcium silicate-based sealers were associated with increased expression of markers related to osteogenic and cementogenic differentiation in hPDLSCs [30,31,33,37,39]. Studies documented consistent upregulation of key markers associated with this differentiation, including ALP, an early marker of osteoblast activity and matrix maturation [27,30,39]; RUNX2, a critical transcription factor involved in early-stage osteoblastic differentiation [27,32,39]; and CEMP1 and CAP, markers associated with cementogenic differentiation and cellular responses potentially relevant to periodontal repair [30,31,39].

Discussion

Chemomechanical disinfection and 3D filling of the root canal system are essential components of clinical endodontics for the success of root canal treatment. The main role of an endodontic sealer is to minimise fluid leakage between the core filling material and the dentinal walls by sealing voids. The root canal system is not compartmentalised, however; it communicates with the periodontium through the apical foramen and accessory canals. Historically, biological inertness of the sealing material was a major focus; with the development of bioactive endodontic materials, this has evolved towards materials that can interact dynamically with the surrounding tissues. In this systematic review, 15 in vitro studies involving hPDLSCs and calcium silicate-based endodontic sealers were reviewed. In general, the data suggest that bioceramic sealers, including BioRoot RCS, TotalFill BC, and CeraSeal, have more favourable in vitro biological characteristics compared with epoxy resin-based materials such as AH Plus. Overall, the data indicate that the chemical composition and ion release of these materials could affect cellular responses relevant to periapical repair. Previous systematic reviews also support the generally favourable biological profile of calcium silicate-based sealers, although findings vary according to material type and experimental conditions [40], while calcium silicate-based biomaterials have demonstrated adequate cytocompatibility and favourable osteogenic/cementogenic differentiation and mineralisation potential in PDLSCs [14].

The Cellular Safety Profile

There were significant differences in cytotoxicity between epoxy resin and calcium silicate-based sealers. Generally, cell viability and proliferation were lower with AH Plus than with calcium silicate-based materials such as BioRoot RCS, C-Root, and BioRoot Flow. A reduction in viable cells at later time points was seen with AH Plus, while calcium silicate-based materials generally showed a time-dependent increase in cell growth. However, there were also variations among calcium silicate-based formulations, with NeoSEALER Flo reducing mitochondrial activity significantly at the tested dilutions, showing that not all bioceramics have the same safety profile, as reported by López-García S et al. (2024) [30].

Overall toxicological findings indicate that most sealers have some level of in vitro cytotoxicity, particularly at high extract concentrations and in the fresh (undried) state, and that bioactive/calcium silicate-based sealers are generally less cytotoxic than epoxy- and zinc oxide-eugenol-based sealers, although outliers such as MTA Fillapex and some experimental compositions have been reported by Fonseca DA et al. (2019) [40] and Silva Almeida LH et al. (2017) [41].

Mineralisation

Mineralised nodules and mineralisation of the extracellular matrix (ECM) were found to be significantly higher in groups treated with materials including TotalFill BC Sealer, Bio-C Sealer, and NeoSEALER Flo than in their respective control groups and, when assessed, compared with conventional resin-based sealers, as reported by López-García S et al. (2019) [28], López-García S et al. (2024) [30], Mora A et al. (2024) [31], and Pérez-Guzmán N et al. (2026) [33]. The calcium and silicon ions released from these materials could stimulate apatite nucleation and mineral deposition, which may be relevant to biological sealing, as reported by López-García S et al. (2019) [28] and López-García S et al. (2024) [30].

In summary, the aforementioned in vitro studies suggest that calcium silicate-based sealers are not only cytocompatible but can also modulate the behaviour of hPDLSCs related to mineralisation, hard-tissue formation, and potentially beneficial immunomodulatory effects relevant to periapical repair processes, as reported by Rodríguez-Lozano FJ et al. (2017) [34], Sanz JL et al. (2021) [37], and Pérez-Guzmán N et al. (2026) [33].

Clinical Implications

The studies do not explicitly address clinical outcomes but rather report bioactivity as a possible criterion of biological sealing. Higher bioactivity may indicate a greater potential to support cellular processes associated with cementum and bone formation at the apical region [34], as well as osteogenic differentiation-related responses in stem cells [33] and an anti-inflammatory environment that may support healing, as noted for BioRoot RCS, CeraSeal, and TotalFill BC by Gaudin A et al. (2020) [26], Mora A et al. (2024) [31], and Pérez-Guzmán N et al. (2026) [33].

To conclude, these in vitro findings suggest that bioactive sealers may support cellular processes relevant to periapical repair; however, they do not establish the formation of a permanent biological seal or superior clinical healing.

The data from the literature review and large-scale evidence syntheses suggest that, despite these mechanistic benefits, calcium silicate-based sealers have not yet been proven to have better long-term clinical performance than conventional sealers; overall, the healing rates of these sealers are similar with regard to clinical outcomes, and the evidence of bioactivity is more convincing at the cellular and tissue levels than from a dramatic improvement in tooth-level outcomes, as reported by Fonseca DA et al. (2019) [40] and Sanz JL et al. (2021) [14].

Strengths and Limitations

This systematic review covers recent premixed CS sealers, dual extraction approaches, and a structured synthesis of biological patterns and mechanisms. It provides a comprehensive review of in vitro studies evaluating the bioactivity of calcium silicate-based endodontic sealers; however, several limitations should be noted. First, most included studies relied on two-dimensional hPDLSC culture models, which were unable to reproduce the complex periapical microenvironment, including interactions with surrounding cells and tissues, immune cell infiltration, and dynamic fluid flow. This simplification may overestimate the cytotoxicity of fresh extracts and underestimate long-term bioactivity under clinically realistic extrusion conditions. Second, methodological heterogeneity, including variability in extract preparation (fresh or set extracts and different surface area-to-volume ratios), dilution protocols, and outcome measures, prevented quantitative meta-analysis and required a narrative synthesis that may be more subject to interpretive variation. Third, small sample sizes and unclear risk of detection bias in several studies due to insufficient reporting of blinding procedures reduce precision and generalisability, as reflected in the modified OHAT assessments. Finally, selection of favourable time points and concentrations may introduce bias, and there are limited data on long-term adverse effects and the biological effects of formulations containing different radiopacifying agents, such as zirconium-, bismuth-, aluminium-, or tantalum-containing compounds. Future studies should use more clinically representative 3D models, standardised extraction and exposure protocols, larger experimental samples, and longer follow-up periods to assess both sustained bioactivity and potential adverse effects.

Future Research Directions

To advance bioactivity assessment of calcium silicate sealers towards clinical translation, standardised in vitro protocols are needed. Future studies should follow ISO 10993-5 and ISO 10993-12 for cytotoxicity testing and extract preparation [19,20], clearly distinguish between fresh and fully set materials, and use predefined and transparently reported surface area-to-volume ratios and dilution series, with core outcomes assessed at predefined endpoints: viability (72 h), migration (72 h), mineralisation (21 days), and a minimal gene panel (ALP and RUNX2 at day 7; CEMP1 and CAP at day 14). Blinded, multi-donor assessments with adequately justified numbers of biological and technical replicates would enhance robustness. Advanced models, including organotypic 3D co-cultures incorporating endothelial and immune cells, microfluidic perfusion systems simulating root canal leakage, and clustered regularly interspaced short palindromic repeats (CRISPR)-edited PDLSCs for mechanistic investigation, offer greater physiological relevance. Comparative evaluations of different radiopacifier systems, including zirconium-, bismuth-, aluminium-, and tantalum-containing formulations, and novel additives under hypoxic or inflammatory conditions are needed, alongside transcriptomic and proteomic profiling to elucidate ion release-dependent pathways. Ultimately, standardised in vitro testing frameworks should inform prospective clinical trials correlating sealer extrusion volumes with periapical healing outcomes assessed using cone-beam computed tomography (CBCT), thereby bridging the translational gap.

## Conclusions

Periapical regeneration has emerged as an important biological consideration alongside the prevention of microleakage. The focus of this review is on hPDLSCs. With their natural ability to differentiate into osteoblasts and cementoblasts in response to molecular cues, these multipotent cells act as an important cellular component of repair. Contemporary tricalcium silicate formulations may influence hPDLSC behaviour through surface topography and ionic release. These materials have shown favourable in vitro bioactivity, biomineralisation capacity, and cytocompatibility; however, their long-term clinical effectiveness remains to be established. Further clinical evidence is required to determine whether these biological effects translate into improved periapical healing.

## References

[1] Kassebaum NJ, Bernabé E, Dahiya M, Bhandari B, Murray CJ, Marcenes W (2015). Global burden of untreated caries: a systematic review and metaregression. J Dent Res.

[2] Wang X, Xiao Y, Song W, Ye L, Yang C, Xing Y, Yuan Z (2023). Clinical application of calcium silicate-based bioceramics in endodontics. J Transl Med.

[3] Janini AC, Bombarda GF, Pelepenko LE, Marciano MA (2021). Antimicrobial activity of calcium silicate-based dental materials: a literature review. Antibiotics (Basel).

[4] Muliyar S, Shameem KA, Thankachan RP, Francis PG, Jayapalan CS, Hafiz KA (2014). Microleakage in endodontics. J Int Oral Health.

[5] Guo J, Peters OA, Hosseinpour S (2023). Immunomodulatory effects of endodontic sealers: a systematic review. Dent J (Basel).

[6] Jafari F, Jafari S (2017). Composition and physicochemical properties of calcium silicate based sealers: a review article. J Clin Exp Dent.

[7] Torabinejad M, Watson TF, Pitt Ford TR (1993). Sealing ability of a mineral trioxide aggregate when used as a root end filling material. J Endod.

[8] Raghavendra SS, Jadhav GR, Gathani KM, Kotadia P (2017). Bioceramics in endodontics - a review. J Istanb Univ Fac Dent.

[9] Song W, Li S, Tang Q, Chen L, Yuan Z (2021). In vitro biocompatibility and bioactivity of calcium silicate‑based bioceramics in endodontics (Review). Int J Mol Med.

[10] Han L, Okiji T (2013). Bioactivity evaluation of three calcium silicate-based endodontic materials. Int Endod J.

[11] Hoshino RA, Delfino MM, da Silva GF, Guerreiro-Tanomaru JM, Tanomaru-Filho M, Sasso-Cerri E, Cerri PS (2021). Biocompatibility and bioactive potential of the NeoMTA Plus endodontic bioceramic-based sealer. Restor Dent Endod.

[12] Lim M, Jung C, Shin DH, Cho YB, Song M (2020). Calcium silicate-based root canal sealers: a literature review. Restor Dent Endod.

[13] Estivalet MS, de Araújo LP, Immich F, da Silva AF, Ferreira NS, da Rosa WL, Piva E (2022). Bioactivity potential of bioceramic-based root canal sealers: a scoping review. Life (Basel).

[14] Sanz JL, Guerrero-Gironés J, Pecci-Lloret MP, Pecci-Lloret MR, Melo M (2021). Biological interactions between calcium silicate-based endodontic biomaterials and periodontal ligament stem cells: a systematic review of in vitro studies. Int Endod J.

[15] Santiago MC, Henrique de Oliveira Salles G, Gomes de Lima G, Alves de Oliveira L, Salles LP (2025). Comparative analysis of viability, proliferation, and mineralization potential of human pulp and osteoblastic cells exposed to different bioceramic endodontic sealers. J Oral Biol Craniofac Res.

[16] Higgins JPT, Thomas J (2024). Cochrane Handbook for Systematic Reviews of Interventions version 6.5. https://training.cochrane.org/handbook/current.

[17] Page MJ, McKenzie JE, Bossuyt PM (2021). The PRISMA 2020 statement: an updated guideline for reporting systematic reviews. BMJ.

[18] Schardt C, Adams MB, Owens T, Keitz S, Fontelo P (2007). Utilization of the PICO framework to improve searching PubMed for clinical questions. BMC Med Inform Decis Mak.

[19] (2026). International Organization for Standardization: Biological Evaluation of Medical Devices-Part 5: Tests for In Vitro Cytotoxicity (ISO 10993-5:2009). https://www.iso.org/standard/36406.html.

[20] (2026). International Organization for Standardization: Biological Evaluation of Medical Devices-Part 12: Sample Preparation and Reference Materials (ISO 10993-12:2021). https://www.iso.org/standard/75769.html.

[21] Nagendrababu V, Murray PE, Ordinola-Zapata R (2021). PRILE 2021 guidelines for reporting laboratory studies in Endodontology: a consensus-based development. Int Endod J.

[22] (2026). National Toxicology Program: OHAT Risk of Bias Rating Tool for Human and Animal Studies. https://ntp.niehs.nih.gov/whatwestudy/assessments/noncancer/riskbias.

[23] McGuinness LA, Higgins JP (2021). Risk-of-bias VISualization (robvis): an R package and Shiny web app for visualizing risk-of-bias assessments. Res Synth Methods.

[24] Campbell M, McKenzie JE, Sowden A (2020). Synthesis without meta-analysis (SWiM) in systematic reviews: reporting guideline. BMJ.

[25] Collado-González M, García-Bernal D, Oñate-Sánchez RE (2017). Biocompatibility of three new calcium silicate-based endodontic sealers on human periodontal ligament stem cells. Int Endod J.

[26] Gaudin A, Tolar M, Peters OA (2020). Cytokine production and cytotoxicity of calcium silicate-based sealers in 2- and 3-dimensional cell culture models. J Endod.

[27] Jing Y, Gong T, Duan C, Wang H, Zhang C, Neelakantan P (2020). In vitro cytocompatibility and osteogenic potential of calcium silicate-based dental cements in a root canal-filling model. J Int Med Res.

[28] López-García S, Pecci-Lloret MR, Guerrero-Gironés J (2019). Comparative cytocompatibility and mineralization potential of Bio-C Sealer and TotalFill BC Sealer. Materials (Basel).

[29] López-García S, Myong-Hyun B, Lozano A (2020). Cytocompatibility, bioactivity potential, and ion release of three premixed calcium silicate-based sealers. Clin Oral Investig.

[30] López-García S, Sánchez-Bautista S, García-Bernal D (2024). Premixed calcium silicate-based ceramic sealers promote osteogenic/cementogenic differentiation of human periodontal ligament stem cells: a microscopy study. Microsc Res Tech.

[31] Mora A, García-Bernal D, Rodríguez-Lozano FJ (2024). Biocompatibility, bioactivity and immunomodulatory properties of three calcium silicate-based sealers: an in vitro study on hPDLSCs. Clin Oral Investig.

[32] Oh H, Kim E, Lee S (2020). Comparison of biocompatibility of calcium silicate-based sealers and epoxy resin-based sealer on human periodontal ligament stem cells. Materials (Basel).

[33] Pérez-Guzmán N, García-Rios P, Rodríguez-Lozano FJ, García-Bernal D, El Yahyaoui A, López-García S (2026). Ultrastructural and immunobiological responses of human periodontal ligament stem cells to novel tricalcium silicate sealers. Dent Mater.

[34] Rodríguez-Lozano FJ, García-Bernal D, Oñate-Sánchez RE, Ortolani-Seltenerich PS, Forner L, Moraleda JM (2017). Evaluation of cytocompatibility of calcium silicate-based endodontic sealers and their effects on the biological responses of mesenchymal dental stem cells. Int Endod J.

[35] Rodríguez-Lozano FJ, López-García S, García-Bernal D (2020). Chemical composition and bioactivity potential of the new Endosequence BC Sealer formulation HiFlow. Int Endod J.

[36] Saber S, Raafat S, Elashiry M, El-Banna A, Schäfer E (2023). Effect of different sealers on the cytocompatibility and osteogenic potential of human periodontal ligament stem cells: an in vitro study. J Clin Med.

[37] Sanz JL, López-García S, Lozano A (2021). Microstructural composition, ion release, and bioactive potential of new premixed calcium silicate-based endodontic sealers indicated for warm vertical compaction technique. Clin Oral Investig.

[38] Sanz JL, López-García S, García-Bernal D, Rodríguez-Lozano FJ, Forner L, Lozano A, Murcia L (2024). Comparative bioactivity and immunomodulatory potential of the new Bioroot Flow and AH Plus Bioceramic sealer: an in vitro study on hPDLSCs. Clin Oral Investig.

[39] Ye Y, Hosseinpour S, Wen J, Peters OA (2025). In vitro bioactivity and cytotoxicity assessment of two root canal sealers. Materials (Basel).

[40] Fonseca DA, Paula AB, Marto CM (2019). Biocompatibility of root canal sealers: a systematic review of in vitro and in vivo studies. Materials (Basel).

[41] Silva Almeida LH, Moraes RR, Morgental RD, Pappen FG (2017). Are premixed calcium silicate-based endodontic sealers comparable to conventional materials? A systematic review of in vitro studies. J Endod.

